# Surface roughness of composite resins subjected to brushing with whitening toothpastes: an *in vitro* study

**DOI:** 10.1590/1807-3107bor-2025.vol39.006

**Published:** 2025-01-20

**Authors:** Nicolle Madruga Ramos FERREIRA, Vinicius Funghetto LIPPERT, Amanda Baptista da Silva HECK, Ana Maria SPOHR, Marcel Ferreira KUNRATH, Carlos Alberto FELDENS, Paulo Floriani KRAMER

**Affiliations:** (a) Cesuca Centro Universitário, School of Dentistry, Department of Dentistry, Cachoerinha, RS, Brazil.; (b) Pontifícia Universidade Católica do Rio Grande do Sul – PUC-RS, School of Health and Life Sciences, Department of Dentistry, Porto Alegre, RS, Brazil.; (c) Pontifícia Universidade Católica do Rio Grande do Sul – PUC-RS, School of Health and Life Sciences, Department of Pediatric Dentistry, Porto Alegre, RS, Brazil.; (d) Universidade Federal do Rio Grande do Sul - UFRS, School of Dentistry, Department of Preventive and Social Dentistry, Porto Alegre, RS, Brazil.

**Keywords:** Composite Resins, Dentifrices, Surface Properties

## Abstract

The emergence of toothpastes containing different abrasive and whitening substances has been a constant concern among dental professionals. The aim of the present study was to perform an *in vitro* assessment of the surface topography of nanoparticle composite resins subjected to simulated brushing with dentifrices. Test samples were prepared with Filtek Universal (3M ESPE), Filtek Bulkfill (3M ESPE) and Z350 (3M ESPE), with 24 samples per resin. A testing machine was used to simulate brushing with the dentifrices Colgate Total 12, Oral B 100% and Oral B Gengiva Detox Gentle Whitening (8 samples per group). The constant speed of the machine was 250 cycles per minute, and 20.000 cycles were carried out, which corresponds to 24 months (1 hour and 20 minutes). Roughness features and qualitative surface topography were investigated. Statistical analysis involved the Kruskal–Wallis, Wilcoxon and Mann–Whitney tests. A significant increase in surface roughness was found for all the resins (p < 0.05). However, no significant difference was found among the resins in terms of final roughness values (p = 0.690). In contrast, a significant difference among dentifrices was found with respect to roughness measurements (p < 0.001). The qualitative analysis revealed an increase in surface roughness in all the samples and differences in the abrasive potential of the dentifrices. In conclusion, brushing with dentifrices increases the surface roughness parameters of composite resin restorations. Moreover, the differences in the abrasive effects of the dentifrices indicate a need for further studies to establish efficacy and safety criteria.

## Introduction

The growing concern for smile esthetics has led to the notion that bright teeth are equivalent to health and beauty.^1,2^ Thus, composite resins have become the material of choice for conservative and aesthetic restorations and are widely used in dental practice for both anterior and posterior teeth.^3,4^

Social networks, associated media and advertising vehicles enhance the aesthetic desire for white teeth. In the last ten years, dentifrices have become more specialized in therapeutic and cosmetic functions, incorporating whitening products with abrasive action.^5^

Relative dentin abrasivity (RDA) was the first parameter used to determine the abrasive potential of toothpastes.^6^ However, manufacturers are not obligated to disclose RDA values, even though the literature reports the need for an acceptably low value to prevent tooth surfaces from being affected by daily dentifrice use.^7,8^RDA values should not be used to rank toothpastes but rather to consider them suitable within an established range.^9^

While whitening toothpastes is essentially limited to the surface, wear on tooth surfaces and restorative materials caused by abrasive forces has been the cause of concern in the dental field.^7^ Over the years, changes have been made in the composition of materials to obtain restorations with better color stability, greater wear resistance and greater surface smoothness. A rough surface increases the retention of plaque and could lead to a reduction in gloss as well as an increase in surface discoloration, which can affect the aesthetic quality and longevity of restorations.^8,10,11^ Consequently, surface properties such as gloss, roughness and wear resistance vary among different composite resin restorations.^11^

The wear of composite resin restorations is related to the degradation of the organic matrix and the loss of clusters of inorganic particles.^10^ Thus, differences may affect the wear resistance of a material, especially with respect to filler content, size, shape and hardness.^11,12^

Based on these new concepts, new classes of composites have been developed in recent years. One example is nanofiller materials containing nanometric particles and nanoclusters or even nanohybrid composites containing ground glass fillers and nanoparticles. Nanocomposites combine the mechanical strength of hybrids and the superior polishing of microparticles, providing better optical characteristics and a reduction in polymerization contraction.^13^

Surface roughness is a set of irregularities formed by numerous grooves and scratches, varying in shape, direction and depth, that arise from interactions with wear processes. Studies have revealed that an average roughness of 0.2 µm is critical for avoiding bacterial adhesion.^14,15^ In another study, the authors reported that a change in surface roughness on the order of 0.3 µm can be detected by the tip of the tongue.^16^

Wear and surface roughness tests after simulated brushing are indicated to analyze the mechanical characteristics of the surface of restorative materials.^8,17^ Studies suggest that composite resins with smaller, more homogeneous particles, such as nanoparticles, have less loss of gloss and greater surface smoothness than resins with larger distinct particles.^17-19^

Although some studies have assessed the effects of brushing different restorative materials,^20-22^ the effects of abrasiveness by whitening toothpastes on the gloss and roughness of composite resins are largely unknown. Knowledge of the abrasiveness of different toothpastes and their recommendations can assist in guiding patients. Therefore, the aim of the present study was to investigate surface roughness modifications on nanoparticle composite resins subjected to simulated brushing with different dentifrices. Thus, the hypothesis of the present study was that a significant difference would be found in surface roughness among the different dentifrices, regardless of the resin used.

## Methods

### Sample preparation and groups

An *in vitro* laboratory study was conducted at the Research and Sample Preparation Laboratory of the Dentistry Course of the School of Health and Life Sciences of *Pontifícia Universidade Católica do Rio Grande do Sul, Brazil* (PUCRS). The samples were made using a bipartite Teflon matrix containing a central opening 6 mm high by 5 mm in diameter, where the composite resin was inserted in 2-mm increments. Twenty-four samples per composite resin were prepared and photoactivated with a blue-phase N device (Ivolcar, Vivadent, Schaan, Liechtenstein) for 20 s each. The energy intensity was measured every five samples with the aid of a radiometer (Demetron, Danbury, CT, USA). Test samples were prepared from discs of the following composite resins (color: A1): Filtek Universal, Filtek Bulkfill and Z350 (Table 1). Surface smoothness was obtained by polymerizing the composite resin cylinder against a polyester strip, which was pressured against the surface of the composite with the aid of a glass plate. The toothpastes Colgate Total 12 Clean Mint, Oral B 100% and Oral B Gingiva Detox Gentle Whitening were selected for the abrasiveness tests (Table 2). Eight samples of each composite resin were distributed for each toothpaste (24 samples for each toothpaste). The samples were thoroughly cleaned in an ultrasonic bath with distilled water for 10 minutes. During the experiments, the samples were stored in plastic compartments containing distilled water.

**Table 1:** Description of the composite resins used in the study.

Variable	Manufacturer	Organic phase	Inorganic phase	Lot
Filtek Universal	3M ESPE	AUDMA, AFM,	58.4% in load volume	2135000332
		diurethane-DMA and 112-dodecane-DMA		
Filtek Bulkfill	3M ESPE	AUDMA,	58.4% in load volume	NF11317
		Dimethacrylate AFM, UDMA, DDMA		
Z350	3M ESPE	Bis-GMA, Bis- EMA, UDMA, TEGDMA,	59.9% in load volume	2208800346
		zirconia and silica		

**Table 2:** Description of dentifrices used in the study.

Brand name	Composition	Manufacturer	RDA
Colgate Tota L 12 Clean Mint	1450 ppm of fluoride, 0.3% triclosan, water, sorbitol, silicon dioxide, sodium lauryl sulfate, PVM/MA copolymer (GantrezTM), aroma, carrageenin, sodium hydroxide, titanium dioxide, saccharine and sodium fluoride.	Colgate-Palmolive, São Bernardo do Campo, SP, Brazil	70–78
Oral B 100%	Sodium fluoride (1450 ppm), water, sorbitol, silica, sodium lauryl sulfate, aroma, carrageenin, sodium gluconate, xanthan gum, zinc citrate, saccharin, sodium hydroxide, limonene	P&G – São Paulo, SP, Brazil	102
Oral B Gengiva Detox & White – Gentle Whitening	Stannous fluoride (1100 ppm), water, sorbitol, silica, sodium lauryl sulfate, aroma, carrageenin, sodium gluconate, xanthan gum, zinc citrate, stannous chloride, stannous fluoride, saccharin, sodium hydroxide, CI77891(titanium dioxide), sucralose, limonene	P&G – São Paulo, SP, Brazil	200

### Abrasion protocol

A simulated brushing machine designed by IDEIA – Research and Development Institute (PUCRS, Porto Alegre, Brazil) was used for abrasion analysis following the protocol described by Chimello et al.^23^ Each test specimen was set in a central hole of an acrylic plate (55 × 25 × 4 mm) with the test surface 1 mm beyond the edge of the hole. The attachment of the sample was performed by applying utility wax for stabilization. Each plate was placed in an acrylic trough fixed to the brushing machine with metal pins. The brushing machine promotes cyclical, rectilinear and bidirectional movements with four jointed arms simultaneously. Each arm had a hole at the far end to fit a rod to which the active head of the dental toothbrush was attached. The total path of movement was 12 mm. Six grams of dentifrice was weighed on a digital scale (Kokay – LM, Brazil) and mixed with 6 ml of distilled water. A homogeneous paste was formed and released into the acrylic trough, completely covering the test samples. The paste and brushes were changed every cycle. The constant speed of the machine was 250 cycles per minute. According to Yilmaz et al.,^24^ 20,000 cycles correspond to 24 months of simulated brushing, which takes 1 hour and 20 minutes. The weight placed on the arm was 200 g, simulating oral hygiene conditions. After the 24-month brushing period, the test samples were washed with tap water, cleansed in an ultrasonic device for five minutes and dried with compressed air.

### Surface roughness

To investigate the surface roughness of the resin discs, an SJ 201 roughness meter (Mitutoyo, Kanagawa, Japan) with an 0.8-mm cutoff and speed of 0.25 mm/s was used for the determination of the surface roughness parameter (Ra-µm). Three readings were taken for each sample at the far ends and center of the sample, guided by markings on an acrylic plate. The needle-shaped stylus of the roughness meter traveled the surface of the test samples with limited movement of 2.5 mm, and the results were digitally recorded in micrometers (µm). The roughness meter was coupled to a metallic base to eliminate unwanted vibrations and ensure accurate readings. The arithmetic average of three readings was used for the analysis. The mean and standard deviation roughness values were determined for each resin before and after the experiment, along with the resulting mean difference and 95%CI. These measurements were also determined for each dentifrice used.

### Qualitative surface morphology

One test sample was randomly selected from each group, and the central area of each sample was chosen for qualitative analysis of the surface topography using scanning electron microscopy with two magnifications (SEM, *Inspect F50 – FEI, Canada*). The samples were dried for 48 h in an environment containing silica gel and kept for 8 h in a low-vacuum environment to remove residual moisture. The samples were then coated with gold/palladium alloys under high vacuum (*Q150RPlus–Quorum, United Kingdom*) for subsequent examination via SEM at magnifications of 200x and 500x.

### Statistics

Considering the asymmetrical distribution of the outcome variable, the Kruskal–Wallis test was used to compare surface roughness values before and after the experiment and the differences found among the composite resins and dentifrices. Considering the dependency of the compared samples, the Wilcoxon test was used to compare the pre- and post-test measurements in the same category of composite resin and dentifrice, and the Mann–Whitney test was used for pairwise comparisons among the dentifrices considering differences in surface roughness. Differences were considered statistically significant when the p value was < 0.05.

## Results

### Roughness assessment

Table 3 displays the surface roughness (Ra) values of the three composite resins before and after being subjected to simulated brushing, irrespective of the toothpaste used. The mean and standard deviation values and differences among the means are highlighted for the comparison of the composite resins. No difference in surface roughness was found among the resins (Filtek Universal, Filtek Bulk and Z 350, St. Paul, USA) prior to testing. A significant increase in roughness was found in all the samples (p <0.05) after the application of abrasive stress, but no significant difference was found among the different resins with respect to the final roughness values (p = 0.690) or the resulting differences (p = 0.733).

**Table 3:** Surface roughness measurements of three composite resins before and after simulated brushing and the resulting differences irrespective of the dentifrice used.

Resin	Initial			Final			Difference		
	Mean	(SD)	p-value	Mean	(SD)	p-value	Mean	95%CI	p-value
All	0.37	0.25		0.75	0.38		0.38	(0.29-0.47)	
Filtek	0.36^a^	0.34	0.395	0.78^b^	0.36	0.690	0.41^A^	(0.23-0.60)	0.733
Universal									
Filtek	0.41^a^	0.25		0.72^b^	0.27		0.31^A^	(0.17-0.46)	
Bulkfill									
Z 350	0.34^a^	0.25		0.74^b^	0.48		0.41^A^	(0.25-0.56)	

SD: standard deviation; CI: confidence interval. Different lowercase letters on the same line denote significant differences before and after treatment (p < 0.05). The same uppercase letters in the same column indicate the absence of significant differences among the resins (p≥ 0.05).

Table 4 displays the surface roughness of the composite resins before and after simulated brushing using the different toothpastes, irrespective of the composite resin used. The mean and standard deviation values and the differences among means are highlighted for the comparison of the toothpastes. The results revealed that the toothpastes used on the resin discs did not differ in terms of surface roughness prior to testing (p = 0.234). After the experiment, however, all toothpastes led to a significant increase in the surface roughness of the resins (p < 0.05), with different final measurements (p = 0.001). Moreover, a significant difference in surface roughness was detected among the different dentifrices. The pairwise comparisons revealed that the roughness resulting from the Oral B Gingiva Detox Gentle Whitening dentifrice was significantly greater than that obtained when the Colgate Total 12 (p = 0.049) and Oral B 100% (p < 0.001) toothpastes were used, whereas the surface roughness generated by the Colgate Total 12 dentifrice was significantly greater than that when the Oral B 100% dentifrice was used (p = 0.027).

**Table 4:** Surface roughness measurements of three composite resins before and after simulated brushing and the resulting differences according to the dentifrice used.

Dentifrices	Initial			Final			Difference		
	Mean	SD	p-value	Mean	SD	p-value	Mean	95%CI	p-value
All	0.37	0.28		0.75	0.38		0.38	(0.29-0.47)	
Colgate total12	0.40^a^	0.29	0.234	0.77 ^b^	0.33	0.001	0.38 ^B^	(0.20-0.55)	0.000
Oral B 100%	0.39 ^a^	0.24		0.55 ^b^	0.26		0.15 ^C^	(0.07-0.25)	
Oral B Detox	0.32 ^a^	0.31		0.92 ^b^	0.43		0.61 ^A^	(0.45-0.76)	

SD: standard deviation; CI: confidence interval. Different lowercase letters on the same line denote significant differences before and after treatment (p < 0.05). Different uppercase letters in the same column indicate significant differences among toothpastes (p ≥ 0.05).

Table 5 displays the differences in the surface roughness of the three composite resins before and after simulated brushing stratified by the dentifrice used. The results revealed that the smallest increase in surface roughness was obtained after the use of the 100% Oral B dentifrice, both for the Z350 and Filtek Bukfill resins, whereas the largest increase was observed after the use of the Oral B Detox dentifrice on the Filtek Universal resin.

### Qualitative analysis – SEM

**Figure 1:**
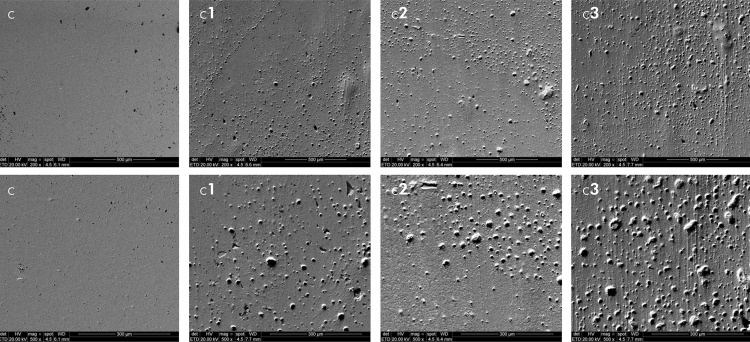
SEM images representative of Filtek Universal (a) resin before brushing. Resin after brushing with OralB 100% (a1); Colgate Total 12 (a2) and OralB Gengiva Detox Gentle Whitening (a3). Magnification 200x (first line) and 500x (second line).

**Figure 2:**
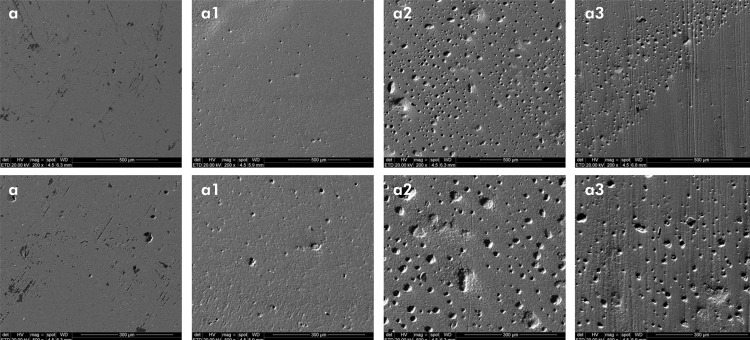
SEM images representative of Filter Bulkfill (b) resin before brushing. Resin after brushing with OralB 100% (b1); Colgate Total 12 (b2) and OralB Gengiva Detox Gentle Whitening (b3). Magnification 200x (first line) and 500x (second line).

**Figure 3:**
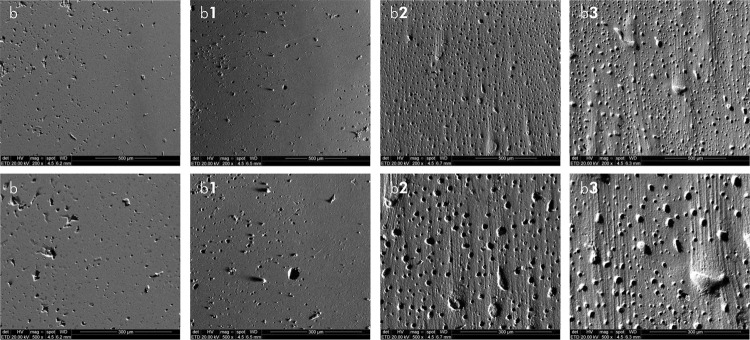
SEM images representative of Z350 (c) resin before brushing. Resin after brushing with OralB 100% (c1); Colgate Total 12 (c2) and OralB Gengiva Detox Gentle Whitening (c3). Magnification 200x (first line) and 500x (second line).

The analysis of the microphotographs (magnification: 200x and 500x) of the composite resin surfaces subjected to brushing revealed a qualitative correlation with increased surface roughness for all the tested samples. Prior to simulated brushing, smooth and uniform surfaces were observed for all the composite resins applied. Moreover, no visual differences were found among the resins. In contrast, after the simulated brushing, the microphotographs confirmed the significant visual difference in the abrasive potential of the toothpastes, as demonstrated by the intense visual modification in morphology and the presence of scratches (Figures 1, 2, and 3). Additionally, after increased magnification via SEM, the surface morphology characteristics changed for all the resins tested, which was correlated with the quantitative roughness results.

## Discussion

Dentistry has made progress in the development of composite resins to obtain restorations with better color stability over time, greater wear resistance and clinically acceptable surface smoothness.^25^ However, there has been constant concern among dentists with respect to the evolution of toothpastes containing different abrasive and whitening substances. On the basis of these findings, the research hypothesis was confirmed because significant differences in surface roughness were found among the different dentifrices, regardless of the resin used.

Most assessment methods are based on relative dentin abrasivity (RDA) or changes in surface roughness following *in vitro* simulated brushing with different toothpastes. It is also important to consider the multifactorial wear of tooth surfaces and restorative materials and that individuals have significant behavioral differences when brushing, which may affect the abrasion potential of a given dentifrice.^9^

Initial surface roughness values are essential for establishing comparison parameters. In the present study, no significant differences were found among the composite resins prior to testing. Moreover, although a significant increase in roughness was found for all the resins after testing, no significant differences were found among the different composite resins. Han et al.^26^investigated the abrasiveness of 20 composite resins in a simulated wear machine and reported no difference in resistance to abrasive wear among nanoparticle composite resins or among nanohybrid resins. In contrast, some studies have reported significant differences among composite resins, especially with respect to inorganic filler content.^27,28^ In the present study, the SEM images revealed an increase in surface roughness for all the resins, with no difference between the materials. The use of composite resins with similar inorganic particles in the present study may have impeded the identification of differences among the materials.

The organic matrix of a composite resin is worn by brushing, which exposes the underlying inorganic particles, as seen in the qualitative analysis in this study. Thus, the restored surface may become rough, leading to an increase in surface discoloration.^29^ Composites filled with nanoparticles have better physical, mechanical, optical, and clinical performance. Nanoparticulate/nanohybrid materials are able to supply a greater load volume with a more homogeneous distribution, which protects the organic matrix from wear and provides a smoother surface.^17,25,30-32^

Irrespective of the RDA values, all toothpastes tested produced a significant increase in surface roughness on all three composite resins. The results also revealed significant differences in the degree of abrasiveness among the different dentifrices. SEM images clearly illustrate the surface roughness of all the samples after brushing and the qualitative differences between the toothpastes used. The roughness resulting from the use of Oral B Gengiva Detox Gentle Whitening was significantly greater than that obtained when Colgate Total 12 and Oral B 100% were used, and the surface roughness resulting from the use of Colgate Total 12 was significantly greater than that resulting from the use of Oral B 100%.

Silica-based abrasives are widely used because of their inert nature. In the present study, all toothpastes contained silica-based particles as the only abrasives; therefore, it was not possible to establish a relationship between the different types of abrasives and the abrasiveness scale. Interestingly, the surface roughness resulting from the use of Colgate Total 12 was significantly greater than that resulting from the use of 100% Oral B, even though the RDA was greater for the 100% Oral B toothpaste. Importantly, the relative dentin abrasivity (RDA) is just one of the parameters used to determine the abrasive potential of toothpastes. Physical characteristics, particularly particle size and shape, are the main aspects associated with the abrasiveness of a toothpaste.^24, 33^ Moreover, the effect of abrasiveness caused by whitening toothpastes on the roughness of composite resins is largely unknown. Thus, the greater surface roughness produced by using Colgate Total 12 (RDA 70-78) in relation to Oral B 100% (102) can be credited, at least in part, to differences in the quantity, size and shape of the particles, water content and degree of agglomeration.

Physical characteristics, particularly particle size and shape,^34^ are the main aspects associated with the abrasiveness of a toothpaste. Under the same application of force, irregular particles produce deeper grooves than rounded particles do, and an identical single particle produces wider, deeper grooves when the force applied is increased. Vicentini et al.^35^ demonstrated that toothpastes with calcium carbonate produce the most intense wear. Joiner et al.^36^ demonstrated that toothpastes containing fine particles of calcium carbonate/perlite or silica presented no significant differences in terms of wear, and Suzuki et al.^13^ demonstrated that calcium carbonate abrasiveness depends on particle size, with smaller particles associated with less abrasiveness. Carneiro et al.^37^reported that the use of a conventional toothpaste with sodium bicarbonate resulted in less wear on the enamel surface than whitening toothpastes did because of the lower hardness of the particles of conventional toothpaste. However, the roughness profile was similar for all the toothpastes tested.

The lack of complete information about the toothpastes may be considered a limitation. Part of this information is included in this paper as revealed by manufacturers; however, the information is limited. Additionally, this experimental study did not reproduce some of the details presented in clinical conditions, such as saliva, temperature, and mouth pH. Therefore, studies using in vivo methodologies and clinical trials must be performed to confirm the statements proposed here.

## Conclusion

Tooth brushing with dentifrices changes the topography of composite resin restorations. A significant increase in surface roughness was found in all the samples, but no significant difference was found among the different composite resins. Additionally, the differences in the abrasive capacity of the different toothpastes were evident. Comparisons revealed that the roughness resulting from the Oral B whitening dentifrice was significantly greater than that obtained when the Colgate Total 12 and Oral B 100% toothpastes were used. The differences in the abrasive effects of the dentifrices indicate the need for further studies using saliva and clinical trials to establish efficacy and safety criteria.
